# Supplementary material for: Elevated psychological distress in undergraduate and graduate entry students entering first year medical school
Source: PLoS One. 2020 Aug 10;15(8):e0237008. doi: 10.1371/journal.pone.0237008 (PMC7416945; doi:10.1371/journal.pone.0237008)
Supplement: Attachment — Submitted filename: manuscript PONE-D-20-04775 review.docx [file pone.0237008.s002.docx]

Comments for Authors

The manuscript covers a relevant topic with an original point of view, and it could be of interest for Plos One’s readers. Method and data analysis are very interesting, however I would suggest some minor changes:

- At page 4, in the section “measures”, please give some psychometric details on the tool, in order that the reader can understand the validity of the measure; is there a specific cut-off for the scale that suggest you to talk about “clinically significant symptoms”? If yes, please describe it better, if not please rephrase the sentences in order to avoid to give judges not based on clinical cut-offs.
- At pag 5, in the Participants’ section, you wrote that 252 students entirely completed the questionnaire.	In order to make it cleare for readers, the percentage should be calculated on the final participants enrolled, so it won’t be 60.9%.
- At pag. 6, when talking about stress in the two different sub-samples, in the text you talk of a p=0.001, while in the table you report p=0.01. Please uniform the values.
- At pag. 6, when discussing Table 2, you write “it can be inferred that around 1 in 3 students has clinically significant depressive and stress symptoms”. I would suggest to change in “depressive and/or stress symptoms”, since we do not know if students with depressive and stress symptoms are the same (this aspect should be replicated also in the discussion section).
- At pag. 7, both in the gender and in the financial sections, you referred to Table 5, while probably you should write “Table 4”.
- At pag. 10, this sentence is not clear: “This makes sense as medicine is their second degree ad so they have been longer in financial debt.”. Please, rephrase it in order to make it clearer than before.
- At pag. 12, please include all the other abbreviations that you use.

Moreover, specific limitations of the study should be considered when discussing conclusions, which should be more cautious and less categorical.

Comments for Editors

The manuscript covers a relevant topic with an original point of view and it could be of interest for Medical Teacher’s readers.

Results and implications are very interesting, however I would suggest to accept the paper with minor changes. In fact, there are different issues that Authors should better explain from the methodological point of view and implications also on the Authors’ conclusions, that should be more cautious and less categorical.
