# Supplementary material for: Elevated psychological distress in undergraduate and graduate entry students entering first year medical school
Source: PLoS One. 2020 Aug 10;15(8):e0237008. doi: 10.1371/journal.pone.0237008 (PMC7416945; doi:10.1371/journal.pone.0237008)
Supplement: Attachment — Submitted filename: Response to reviewers.docx [file pone.0237008.s003.docx]

**Response to reviewers**

Dear Academic Editor Plos One and reviewers,

Thank you for taking the time to edit and review my article. I would like to take the time to address the editor and reviewer notes.

Editor

- I have taken note of the suggestion about the title and have amended the title to a more fitting one.
- I have changed distress to psychological distress to avoid confusion
- I have made changes to the DASS-21 information in the methods, included inclusion and exclusion criteria, and produced a sample size calculation. I have also amended the methods section to explain what constitutes ‘significant’
- I have amended the article to include discussion about confounders and added it to the limitation section
- I have added a section in the discussion about the 40% of students not participating in the research
- In the discussion section, I have talked more specifically about the prevalence in the difficulties associated with this in Australia and also more widely.
- Statistically significant has been amended to significant and other typos have been addressed
- The informed consent information requested has been updated in the methods section
- The table numbers have changed – no tables were missing but mislabelled
- Supporting file caption names have been addressed
- Ethics statement is within the methods section

Reviewer 1

Thank you for your review. I have been through your comments and they have been helpful. I have made the minor changes listed and corrected my P-values. I have attended to the changes suggested and made them on pages 4, 5, 6,7, 10 and 12. I have also re-looked at my conclusion to make it a little less categorical.

Reviewer 2

I have listed the software and listed the study period. I thank you for your review.

Reviewer 3

Thank you for your review. I have made a note of your suggestion in my discussion about lack of information about the reasoning for differences between undergraduate and graduate students psychological distress. I have put some more thought into it and made some of my own inferences based upon the current research.

Sincerely,

Sean Atkinson, FRACGP

Chief investigator

Monash University
