# Supplementary material for: Elevated psychological distress in undergraduate and graduate entry students entering first year medical school
Source: PLoS One. 2020 Aug 10;15(8):e0237008. doi: 10.1371/journal.pone.0237008 (PMC7416945; doi:10.1371/journal.pone.0237008)
Supplement: Attachment — Submitted filename: Response to reviewers 2 (1).docx [file pone.0237008.s004.docx]

**Response to reviewers**

Dear Academic Editor Plos One,

Thank you for taking the time to edit and review my article. I would like to take the time to address the editor minor revisions. There was no reviewer for the minor revisions.

Editor

I have made changes to the tables and put the headings at the top instead of the bottom

I have amended the references – especially reference 14

I have ensured the ethics section is within the methods

I have moved the location of the size calculation to the suggested place in methods

Further – I was requested in the email from today (3/7/2020) to list why the data is not publically available and the names of a point of contact for it if needed etc – however, the data is within the supporting information and I have stated this in the data availability section on the upload website.

I have no concerns about the minor revisions and complied happily.

Sincerely,

Sean Atkinson, FRACGP

Chief investigator

Monash University
